# Editor’s Table

**Published:** 1858-08

**Authors:** 


### EDITOR'S TABLE.

THE NEW AMALGAM is not quite killed off, even by Dr. Townsend's final card, in which (as a kind of counter-balance to the very extravagant flourish of praise with which he first heralded it to the world), he declares that he has abandoned it altogether. Extremes meet, especially foolish ones.

Dr. White declares that, "the blackest teeth he has ever seen, were those filled with the new amalgam."

Now with all deference to Dr. W.'s assertion, it is a fixed fact that *pure* silver, tin and mercury, will produce a less oxidzable compound, than those materials of only *commercial* purity, when there is present copper and lead, and frequently other impurities.

It is also true that much of the material used as the *New Amalgam* is not made of pure metals, nor perfectly prepared either in the melting or filing, nor even in the subsequent manipulations, and yet the results following its use are charged to the *New Amalgam*, and it is judged thereby.

Many of the conservative members of the profession, of our acquaintance, use it and assert that in a certain class of cases with the genuine amalgam they can do better work in every respect, than can be done with gold, and will continue to use it let who will denounce it.

That it is sadly misused there is no doubt; that sensitive teeth are imperfectly cleaned and plastered up with it is too true; but the same class of teeth are quite as imperfectly prepared for filling with gold, and gold fillings are put into such cavities quite as imperfectly as amalgam can be. In either case the teeth become discolored in a short time and the plugs are lost, but it is not the fault of the material in either case.

We are prepared to furnish the "New Amalgam" as originally described in its purity, and feel well assured that proper subsequent manifestations will produce all the results claimed for it by its original inventor.

---

The Dentist is frequently troubled in the course of putting a mouth in order, by chapped lips or excoriations of the corners of the mouth. A friend who has tried it, recommends the use of equal parts, by weight, of Glycerine and Tannin. The Tannin readily dissolves in the Glycerine.

Several cases of hemorrhage following extraction of teeth, some of which were uncontrolled by lunar caustic or tannin, having fallen into my hands I was much pleased by the rapid and complete effect of the new styptic, the sesqui chloride of Iron, and recommend it to those so unfortunate as to have a case of the kind.

—

OHIO COLLEGE OF DENTAL SURGERY.—We take pleasure in calling attention to this institution. Every chair is filled with just *the man for the place.*

The course of instruction is thorough and complete, and every facility afforded for a full and practical inculcation of the knowledge and skill requisite to enable the student to become a first class Dentist. The Professors are all in the prime of life, active, energetic and zealous in the cause. We feel confident that the Dental student can nowhere do so well, nor in any way spend the time and money so profitably as by attending a course of lectures in the "Ohio College of Dental Surgery." See advertisement.

—

ADVERTISEMENTS.—Our space is too limited to notice each advertisement separately, but we trust our readers will examine them all with care, as they are all worthy of notice. Our aim in this department is to have advertisements *only* from reliable establishments, and thus far at least, we have succeeded.

—

### THE BANQUET.

For the gratification of those who were not present we publish the card of invitation and bill of fare of the banquet given by the Dentists of Cincinnati to their brethren from abroad:

THE PLEASURE OF YOUR COMPANY IS SOLICITED
AT A
COMPLIMENTARY ENTERTAINMENT,
TO BE GIVEN
BY THE DENTISTS OF CINCINNATI AND VICINITY,
AT MELODEON HALL.

| | | |
|---|---|---|
| Thursday Evening, 8 o'clock, | CHAS. BONSALL, | |
| August, 1858. | JAS. T. IRWIN, | Committee. |
| | JNO. T. TOLAND, | |

We have in type the Bill of Fare, Wine List, Volunteer Toasts, &c., but our Printer tells us they have been crowded out. We will however add, that the company had a good time, listened to some good speeches, and partook liberally of the good things set before them.

